# Factors affecting women’s decision between uterine-preserving versus hysterectomy-based surgery for pelvic organ prolapse

**DOI:** 10.1177/17455057231181015

**Published:** 2023-06-30

**Authors:** Kaylee Ramage, Ariel Ducey, Natalie V Scime, Erin Knox, Erin A Brennand

**Affiliations:** 1 Department of Obstetrics and Gynecology, Cumming School of Medicine, University of Calgary, Calgary, AB, Canada; 2 Department of Sociology, Faculty of Arts, University of Calgary, Calgary, AB, Canada; 3 Department of Community Health Sciences, Cumming School of Medicine, University of Calgary, Calgary, AB, Canada

**Keywords:** hysterectomy, pelvic organ prolapse, shared decision-making, surgery, uterine preservation

## Abstract

**Background:**

Given the prevalence of women seeking surgical treatment for pelvic organ prolapse (POP), there is a need to understand women’s decision-making regarding uterine-preserving versus hysterectomy-based surgeries. Historically, hysterectomy-based surgeries have been the preferred treatment for pelvic organ prolapse; however, contemporary evidence supports uterine-preserving surgeries as equivalent. At present, the lack of information available to the general public and limited options presented at surgical consultation for pelvic organ prolapse may hinder women’s autonomy as they navigate surgical treatment.

**Objectives:**

To examine the factors affecting women’s decision-making processes regarding uterine-preserving or hysterectomy-based surgery for pelvic organ prolapse.

**Design:**

This is a qualitative study.

**Methods:**

We conducted semi-structured, qualitative interviews with women seeking surgery for pelvic organ prolapse to explore the factors affecting women’s decision-making between hysterectomy-based and uterine-preserving surgeries.

**Results:**

Women (n = 26) used clinical and personal factors to determine which surgery was best. Women noted that the lack of evidence (clinical and/or anecdotal) available to them hindered their decision-making, causing them to rely more on their own interpretations of the evidence, what they perceived to be “normal,” and what their surgeon recommended. Even with standardized discussion regarding the existing clinical equipoise between surgeries at the clinical consultation, some women still had misperceptions that hysterectomy-based surgery would convey the lowest risk of prolapse recurrence and be best for severe prolapse.

**Conclusion:**

There is a need for more transparency in discussions about prolapse and the factors affecting women’s decision-making for surgical repair of pelvic organ prolapse. Clinicians should be prepared to offer the option of hysterectomy-based or uterine-preserving surgeries and to clearly explain the clinical equipoise between these procedures.

## Introduction

Pelvic organ prolapse (POP), when one or more pelvic organs (i.e. the bladder, uterus, small bowel, rectum) descend into or through the vagina,^1^ affects roughly half of parous females over a lifetime.^1–3^ While POP may be asymptomatic, those with symptoms report impact on quality of life due to feelings of pressure or bulging in the pelvis, pain, fecal or urinary incontinence, obstructive voiding, and/or sexual discomfort.^4,5^ Surgery for POP is common, with approximately 12%–19% of women with POP opting for surgery by age 85 years.^6,7^ Traditionally, surgical treatment of apical POP involved a hysterectomy and suspension of the vagina,^8,9^ but with the development of new surgical techniques to treat POP (including minimally invasive vaginal and endoscopic approaches),^9^ the appropriateness of hysterectomy-based surgery has been questioned. The uterus may play a structural role in pelvic floor stability, and thus, uterine-preserving surgeries (UPSs) such as uterine suspension may confer long-term benefit compared to hysterectomy,^8^ although research comparing the two surgeries has shown clinical equipoise with respect to anatomic cure of POP.^10,11^

Recent evidence indicates that women experiencing prolapse may desire to avoid hysterectomy, with more women choosing UPS for POP repair. In a small quantitative study, 60% of women referred for evaluation of prolapse indicated they would decline hysterectomy if presented with an equally effective option.^12^ Similarly, in a US cross-sectional study examining patient preferences for uterine-preserving versus hysterectomy-based surgery in women with prolapse, assuming equal likelihood of success, 36% of women preferred UPS, 20% preferred hysterectomy-based surgery, and 44% indicated they had no preference.^13^ Both these studies were performed in the United States, and it is possible that social, cultural and health systems aspects could impact patient preferences and as such, these studies are worth replicating in other contexts.

Despite women desiring UPS, they are not always given this option during clinical counseling, potentially reflective of low value placed on women’s autonomy in health decision-making. Historically, the etymology of the word “hysterectomy”^14^ maps back to “hysteria” as a mental health disorder used to misdiagnose females for centuries,^15^ with treatment often being surgical excision of the uterus. The fact that this is the only name for a surgical procedure that does not map to an anatomic organ is not only relevant to how women’s health has been viewed in the past but also serves as a modern-day reminder that female bodies are not prioritized in healthcare. Women’s health issues are often ignored, and research to investigate treatment and other supports is perpetually underfunded compared to men’s health issues.^16,17^ There has been a lack of evolution in surgical methods for prolapse, and the continued promotion of the hysterectomy-based approach to POP surgery as default, despite mounting evidence for uterine-preserving techniques,^8^ raises concern that paternalistic practices rooted in the stereotype of “women’s issues” being fixed by hysterectomies has led to continued de-prioritization in empowering women regarding a choice in the surgical treatments that they consider for POP.

Little evidence is available to understand *why* and *how* women decide between hysterectomy-based or uterine-preserving surgery. This paucity of evidence reflects the continued devaluation of research and women’s experiences to inform women’s reproductive health. There is significant evidence indicating that women’ symptoms and experiences in healthcare are often ignored, trivialized, or dismissed by physicians,^18–20^ termed “medical gaslighting,”^21^ and this experience has been documented in treatment-seeking for POP.^22^ Furthermore, although surgical expertise plays a role in determining surgical treatment for POP,^8^ many surgeons do not have the training or expertise to perform uterine-preserving procedures^10^ resulting in a perpetuation of the historical hysterectomy-based approach to POP surgery and limiting women’s bodily autonomy and informed choice in treatment-seeking. In response to this gap in the literature, this study aims to center women’s voices in answering the question: what factors affect women’s choice between hysterectomy-based and uterine-preserving surgeries for treatment of POP?

## Methods

We conducted semi-structured qualitative interviews with a purposive sample of participants who had elected to take part in a prospective cohort of patients undergoing surgical repair of their POP at a women’s health clinic in a large city in Western Canada; the details of which are described elsewhere.^23^ Women were eligible to participate if they had diagnosed apical POP of the uterus ⩾stage 2 as defined by the Pelvic Organ Prolapse-Quantification System,^24^ elected surgical management of POP, had no prior hysterectomy, desired no further pregnancy, could communicate in English, and were at least 18 years of age. In our sampling, the research team aimed to ensure that both uterine-preservation and hysterectomy surgeries were represented among participants, as well as to capture the diverse experiences of women experiencing POP (e.g. diversity of age, reproductive history, prolapse symptoms). As the sampling was done iteratively with the transcription and analysis, sample size was ultimately determined through team decision’s regarding the diversity of the sample and saturation of themes.

Surgical consultations took place at a women’s health clinic at a large, urban hospital in Canada. In Canada, surgery for prolapse repair is covered by the universal healthcare system without cost to patients. In this setting, both approaches to surgical repair of POP are offered, providing the unique opportunity to examine women’s choices between the two surgeries. The initial surgical consultation was conducted with one of three Female Pelvic Medicine and Reconstructive Surgery (FPMRS) specialists who could perform either uterine-preserving or hysterectomy-based surgery POP repair, during which the surgeon would discuss both potential treatment options with the woman. In the absence of concomitant pelvic pathology resulting in a recommendation for a hysterectomy (such as endometrial hyperplasia or cervical dysplasia), all women opting for surgery were presented with a choice between hysterectomy and vaginal vault suspension (i.e. hysterectomy-based surgery) or uterine suspension (i.e. UPS) to address the apical aspect of their POP. Both options were presented in neutral, value free terms using the available clinical evidence (as described in Table 1), with neither option being described as being the “correct” or “best” option, although surgeons might still make recommendations if asked directly by a patient. Evidence indicating clinical equipoise between procedures was discussed.^10^ Pamphlets from the International Urogynecology Association on prolapse and the surgical options for treatment of prolapse were used to standardize discussion.

**Table 1.** Description of standardized counseling regarding hysterectomy-based versus uterine-preserving surgical options.

	Hysterectomy-based surgery	Uterine-preserving surgery
Context of the procedure	Traditional approach to POP surgery once childbearing is complete Over 100 years of this approach being the predominant modality studied in research	Less commonly performed, fewer research studies, shorter follow-up time windows to quote cure Uterus is hypothesized to act as a central structural support in the pelvis
Cure rate	85% objective POP cure for short and medium term	80%–90% objective POP cure short and medium term
Risks	Blood transfusion—1% of cases Rare adverse events related to anesthesia Venous thromboembolism Urinary tract infection—up to 5% Injury to adjacent organs—up to 2% Development of new onset (latent) stress urinary incontinence	Blood transfusion—<1% of cases Rare adverse events related to anesthesia Venous thromboembolism Urinary tract infection—up to 5% Injury to adjacent organs—up to 2% Development of new onset (latent) stress urinary incontinence
Benefits	Results in loss of menses, which may be of benefit for women with problematic menstrual disorders If all prior PAP tests were normal for that individual, no further PAP tests are needed	Shorter operative time Less over all blood loss
Time in hospital	Most women stay one night, small proportion require second or third night in hospital	Most women stay 1 night, small proportion require an additional night in hospital
Recovery time	Approximately 6 weeks of no heavy lifting	Approximately 6 weeks of no heavy lifting
Subsequent prolapse	15% risk of recurrent prolapse Recurrent apical prolapse could be addressed surgically, via: ∘ native tissue vault suspension, or ∘ mesh-based vault suspension	10%–20% risk of recurrent prolapse Recurrent apical prolapse could be addressed surgically, via: ∘ repeat uterine suspension, native tissue or mesh-based ∘ hysterectomy-based POP surgery with vault suspension

POP: pelvic organ prolapse; PAP: Papanicolaou Test, a procedure to collect cells from the cervix, which can be used to assess cell changes, or to test for cervical cancer other conditions.

After their initial surgical consultation, women could take time to decide which surgery they would like. Once they informed the FPMRS care team regarding their decision, their surgery was scheduled. There was no prescribed time period for this decision. Women with surgeries scheduled between February and July 2021 were invited to participate in the interviews. Interviews were conducted by K.R. with participants via Zoom or telephone depending on patient preference and were audio-recorded. Participants could ask clarifying questions before and during the interview. Questions focused on women’s experiences of POP, their journey seeking care for POP, and their decision-making around surgery. Women described their knowledge of the relative risks and benefits of the two surgeries, their feelings about their uterus and its potential loss, and their sources of information regarding options for treatment. Interviews took approximately 45 min.

Interviews were analyzed by K.R. and E.K. thematically using the Framework Method,^25^ a qualitative analysis method especially appropriate for health and health policy research, as it does not adhere to any single theoretical or conceptual ideals^26^ and allows for comparison between study populations (e.g. those electing for uterine-preserving compared to hysterectomy-based surgery). First, each interview was transcribed verbatim and anonymized. Researchers then familiarized themselves with the transcripts by reading and re-reading. Two interviews were coded by both researchers using qualitative analysis software (NVivo 12, QSR International) to develop an analytical framework. Coding discrepancies were discussed and resolved, creating a working analytical framework to apply to the transcripts. Each transcript was then coded with the revised analytical framework. Once the coding was complete, data were charted into a framework matrix, along with summaries of findings for each major theme. Finally, data were interpreted with the larger team (E.A.B., E.K., K.R., N.V.S.) to discuss implications, connections between themes, and triangulation with experiences from clinical practice.

### Reflexivity statement

Our interdisciplinary study team was composed of both clinical and research professionals, with expertise in epidemiology (K.R., N.V.S., E.A.B.), sociology (E.K., A.D.), and urogynecology (E.A.B.). As self-identified women working on women’s health, our research team has insight into the gender roles and experiences of women seeking care and balancing gendered identities when making choices about our own bodies. The interdisciplinary nature of our team allowed for examining multiple interpretations of the qualitative data, linking themes, and data validation and triangulation.

## Results

Twenty-six women participated in the qualitative study, with approximately 54% of opting for a hysterectomy-based surgery (n = 14) and 46% opting for a UPS (n = 12). Median age of participants was 53 years (interquartile range (IQR) = 40–67). One woman initially opted for a UPS but had changed her mind by the time of her interview. Most of women were White and had completed at least some post-secondary education. All participants were biologically female and self-identified as cisgendered women. All except one reported being heterosexual (one woman reported being bisexual). Most women were married or in a common-law relationship.

Women’s decision-making regarding surgery for POP repair was complex, dependent on the information and expertise available to them, their preferences and values, and personal characteristics. Overall, women noted difficulty in making decisions about surgical treatment for POP, given the limited clinical evidence and the general societal taboo they faced when seeking information. Many women had limited understanding of POP and their treatment options prior to their diagnosis; however, many reported knowing that a hysterectomy was likely to be advised. None reported knowledge of UPS prior to their clinical consultation.

Women discussed the limited awareness of prolapse in the public discourse and its impact on their ability to make informed decisions. Other women highlighted the stigma and shame that they felt regarding their symptoms, which limited their ability to talk to others. When women did approach others to discuss their experiences with POP, they found friends or family members provided vague information, as most women with lived experience did not feel comfortable discussing their symptoms with others.

Despite the reported helpfulness of the comprehensive clinical consultation, the standardized discussions, and the information provided to women to take home, many women had unanswered questions about surgery and felt as though more information was required for their surgical decision-making. With the scarcity of the available clinical evidence, women relied on their personal beliefs, their interpretations of the available data presented to them, and their values to decide on the surgery that was best for them. From the interviews, several themes emerged regarding the factors which influenced women’s decision-making between uterine-preserving and hysterectomy-based surgeries: perceived risks and benefits of each surgery, attachment to the uterus, hysterectomy as the default, surgical failure, prolapse severity, role of uterus in pelvic floor stability, and trust in surgeon. Each theme is discussed below; exemplar quotes are available in Table 2.

**Table 2.** Qualitative themes and exemplar quotes.

Theme	Sub-theme	Exemplar quote
Age	Uterine-preserving surgery	“Saving it gives me more options if I [prolapse again] . . . . And to think that I would rob myself of one extra option, it just didn’t make sense. So, I think that was the main reason why I chose the uterine saving surgery, is that it just gives me one more chance.” (Participant 15)
	Hysterectomy-based surgery	“I understand that I’m quite young. But whenever I have periods, they’re quite heavy. [The surgeon] did provide me with an option to get an IUD [to lighten them] but I just didn’t want to keep having to do that until menopause. The [low] recurrence rate of a prolapse was the deciding factor for me as well, because if the rate would have still been high, I probably wouldn’t have done it . . . Like [I’ll be] done after this, one surgery and done. Yeah, that’s my hope. But you never know, whenever I’m like 60 or 70, maybe I’ll need to have it redone.” (Participant 10)
Perceived risks and benefits of each surgery	Uterine-preserving surgery	“The less you disturb things, the better. I guess that would be my approach. So, if it didn’t need to come out, there was no point in going ahead with it.” (Participant 26)
	Hysterectomy-based surgery	“I questioned the risks of the hysterectomy versus no hysterectomy, and [the surgeon’s] addition was the risk of bleeding. And I think in this day and age, that would be a manageable risk.” (Participant 12) “Initially, I decided if I’m going through the surgery, it was lowest risk and the least complicated. So that was the preservation. And then I thought about it and I said, well, if I’m going through all of this, I might as well also remove the uterus and not have to deal with my really annoying periods . . . . And yes, it’ll be a little bit harder on the body. But I’m young and I can recover.” (Participant 14)
Attachment to the uterus	Uterine-preserving surgery	“I’ve always thought of it as not just another organ. It’s the home of my babies. My three babies grew up in there. It’s what makes a woman . . . Without a uterus are you a woman? By definition, no. Like, that’s the female reproductive organ.” (Participant 15) “When [the surgeon] mentioned about taking it out, suddenly it didn’t feel right. I’ve heard of women who had hysterectomies and how they changed afterwards . . . And maybe it’s just psychological . . . It’s very symbolic. And in the past, I would have just poo pooed the whole thing.” (Participant 19)
	Hysterectomy-based surgery	“A lot of people feel it’s part of womanhood. Get rid of it. There’s no reason for it, you know, and also, I’ll have less chance of cancer for it. Take it out of there.” (Participant 6) “I have no attachment. There was a little bit where I was [debating whether to have another child but decided against it] . . . For me, because I made the decision and the decision wasn’t made for me, I am totally at peace with it.” (Participant 2)
Hysterectomy as the “default”	Hysterectomy-based surgery	“[The option] wasn’t presented that there are reasons why [you would keep your uterus], other than attachment [to the uterus]. Other than if it made me feel less like a woman or something, if my uterus [is taken out]. That there’d be any reason to keep it, and whether I had a uterus or not, didn’t seem to matter to me.” (Participant 3) “[Before the consultation], I knew what a prolapse was. My assumption for treatment was just a full-blown hysterectomy. That’s the only way to fix it.” (Participant 4)
Surgical failure	Feeling like uterine-preserving surgeries give an option if prolapse recurred	“But this gives me one more option, right? My next step [if surgery fails], instead of a surgery that could close up my vaginal canal, I can have a hysterectomy then.” (Participant 15)
	Feeling like a hysterectomy-based procedure would prevent prolapse recurrence	“Let’s do this, and let’s make it permanent. Let’s do it. Let’s do it right. I don’t need to try to do this to find out that it’s not going to work . . . then have to go in there again and do the original in the first place. Let’s just do it. Get her done.” (Participant 5) “I decided that . . . if I don’t have a uterus, it’s not going to fall down.” (Participant 3)
	Lack of knowledge of prolapse recurrence even with hysterectomy-based surgery	“I didn’t know. I thought it was like a one and done thing. You do it once and you fix it . . . I had no idea the vaginal vault could prolapse if you get a hysterectomy. . . Yeah, it was very mind opening to learn that it could happen again.” (Participant 15)
	Age as a factor	“I wanted less hassle at my age . . . [the surgeon] explained what all my choices were, and I just did not wish to diddle around with . . . stopgap measures [i.e., a uterine-preserving surgery]. I wanted it done.” (Participant 9)
Prolapse severity	Perceived need for hysterectomy due to prolapse severity	“It’s too prolapsed. [The uterine-preservation surgery] was not going to be satisfactory . . . I didn’t question that. So, let’s do this, and let’s make it permanent. Let’s do it right. I don’t need to try to do this to find out that it’s not going to work.” (Participant 5) “I don’t think I have a choice because I was told that my uterus is slipping too.” (Participant 13)
The role of the uterus in pelvic floor stability	Structural support provided by the uterus	“So, my understanding is that the uterus provides some structure to the stability of the pelvic floor and that’s why I’m keeping it. I understand that my uterus is healthy, so I would like to keep it, I don’t have any fibroids or any problems. So that’s why I’m keeping it.” (Participant 18) “I know it’s a ‘useless’ organ these days, but maybe in the future they’re to discover like. . . they used to take your tonsils out, they used to take your . . . your appendix. I’m of the generation where it was a routine. You just did it before you had trouble with it. I kept both of mine and now they’re discovering your tonsils are used in fighting infection. I have a really good immune system. And they’re now finding uses for [the appendix] as well. So what’s to say that the uterus is not useful after all?” (Participant 19)
	Potential for prolapse recurring without the structural support of the uterus	“I kind of came to the understanding at some point during the conversation that I didn’t need to have a hysterectomy. I could have these repairs with a hysterectomy. But apparently there is research, recent research that is pointing to the fact that that’s not absolutely necessary. And also, there is the . . . I understand that there is some possibility of other things prolapsing once the hysterectomy takes away your uterus.” (Participant 16)
Trust in the surgeon	Uterine-preserving surgery	“I hate to say [that I didn’t consult any other sources]. I just trusted her. I trusted her forthrightness. I trusted her honesty and her knowledge. [She] inspired my confidence.” (Participant 17)
	Hysterectomy-based surgery	“I made the call then and there [during the surgical consult]. And then came home and talked to my husband afterwards. It’s my body. I am not about to consult anyone else. I’ve got a medically trained doctor telling me, assuring me. So that was that. It’s kind of like, you know, you’ve got a broken foot and the doctor wants to put a cast on it. I’m not going to call a friend. Let’s go ahead and cast it.” (Participant 1) “And she seemed very knowledgeable, and I have . . . no fear, or anxiety or . . . Like I trust her completely as far as this procedure.” (Participant 4)
	Decided on their surgery based on the recommendation of the surgeon	“I asked [the surgeon] what she would do if it was her and she said, well, at my age, she would probably just simply have it removed because in the long run it would be less of a hassle. And I didn’t want hassle. I’m [in my 70s] and I didn’t want any more hassle with this. And that’s how I reached the decision to have it removed.” (Participant 9) “I don’t have any strong opinion here. I’m just thinking, what do you recommend? And so when she said that she would recommend [hysterectomy-based surgery], that’s the way we’re going.” (Participant 8)

IUD: Intrauterine device, a form of long-acting contraception.

Of note, the research team was surprised that there was no notable difference in the age of women opting for each approach. For example, two women with similarly young age and who were counseled by the same surgeon chose different surgeries, having different interpretations of the available evidence. One participant, who chose a UPS, noted her attachment to her uterus but also her trust in the surgeon and the available evidence that UPS would be her best long-term option, in case the prolapse recurred. Conversely, the other woman noted that, because the risks presented for the recurrence of prolapse were low, she opted for the hysterectomy-based surgery which she perceived as beneficial due to fact that removal of her uterus would result in amenorrhea. This finding highlights the importance of other factors affecting women’s choice between uterine-preserving or hysterectomy-based surgery.

### Perceived risks and benefits of each surgery

Women in this study appeared to evaluate the risks and benefits of each surgery in their decision-making process. The 12 women, who opted for uterine preservation felt that UPS had less “risk” of surgical complications, was “less invasive,” had a “shorter recovery” time, and had lowered risk of failure in the future. One woman who opted for a hysterectomy-based surgery acknowledged the decreased peri-operative risk associated with UPS but felt that the additional risks associated with hysterectomy were manageable. Other women discussed the perceived benefit of hysterectomy-based surgery as removing their risk for potential uterine cancer or, for women who had not yet reached menopause, addressing heavy and painful menstruation. Ultimately, women used their perception of the risks and benefits to choose the surgery which would align with the outcomes they wanted the most (e.g. not wanting to have to have another surgery in the future).

### Attachment to uterus

Some women who chose UPS discussed their attachment to their uterus, and its relation to their femininity and womanhood, and childbearing. One woman explained her attachment becoming evident to herself only when faced with the decision to preserve or remove her uterus, demonstrating that if only one option is presented, women may not have the opportunity for self-reflection. Women who chose a hysterectomy-based surgery tended to not feel attachment to their uterus, feeling instead that the absence of their uterus would not affect their identity. One woman interpreted that during her clinical encounter that she had not been presented with any specific reasons for keeping the uterus, other than if she felt attached to it. Another stated that she did not feel as though the uterus was related to her identity as a woman. These statements may be related to our sample having completed their families (as a requirement for participation in the study).

### Hysterectomy as the default

Several women mentioned that hysterectomy was the traditional approach to treating prolapse and perceived it to be the logical, default choice when dealing with their POP, based on experiences with friends or family, previous encounters with health professionals, or general societal norms. One woman discussed her perception that she needed a specific reason to detour from a hysterectomy-based surgery. Even if women did not know the full range of surgical treatment for POP before seeing the surgeon, they usually knew about hysterectomy-based surgery as an option. Hysterectomy-based surgery was perceived by most women to be the “normal” surgical approach, which was reinforced by anecdotal evidence from their general family physician, family, and/or friends. The anecdotes given regarding women’s decision-making in favor of hysterectomy seems to reveal a heuristic that allows that navigation of decision-making to be more straightforward for some, as they did need not feel the need to think through all the available evidence. This speaks to the need to address the continuing (mis)perception, even among physicians and gynecologists, that hysterectomy is the best and perhaps only way to surgically address POP as it influences how patients process information and may lead to patients discounting objective facts about other surgical options.

### The role of the uterus for pelvic floor stability

Some women opting for UPS articulated that their understanding of the uterus’ possible role in providing structural stability to the pelvic floor was part of their decision-making process, with one woman expressing that evidence could emerge in the future regarding the utility of the uterus for overall health. Those selecting uterine preservation felt that hysterectomy-based surgeries were not necessarily the best option, conveying the understanding that loss of the uterus could still result in subsequent prolapse of the top of the vagina. One woman felt like the benefits of the UPS outweighed those of the hysterectomy-based surgery, even though she did not have a specific attachment to her uterus.

### Surgical failure

Most women were aware that POP could recur after surgical correction because they had received this information during the surgical consultation. Women who were younger and/or experiencing severe prolapse symptoms were especially aware of the possibility that POP could recur after surgical correction. Women who selected uterine-preserving procedures discussed keeping their uterus so that they would have “another option” for surgery if they did experience recurrence of POP symptoms. In contrast, women who selected hysterectomy-based surgery viewed it as a more definitive solution, with a lower likelihood of failure, contrary to the standardized evidence presented and discussed during consultation.

It is interesting that some women perceived hysterectomy-based surgeries to have a higher cure rate than UPS, especially given the lack of clinical evidence and clinical counseling to this effect. Discussions with women suggested that this idea was related to a bias or misconception that hysterectomy-based surgeries would not fail because the uterus would not be there to prolapse. Women’s interpretations of the available evidence may be affected by their use of heuristics, where women are basing their decision-making not on the evidence presented to them in the clinic setting but on what they have heard or seen anecdotally from other women or from the media.

### Prolapse severity

In recounting their decision-making process, two women felt that prolapse severity dictated their choice for hysterectomy-based surgery, and that UPS was no longer an option. Several women discussed their choice to get a hysterectomy-based surgery being related to the severity of their prolapse, even though there is no clinical evidence to suggest severe POP is better treated by either approach. Internally held misconceptions and misinformation thus limited women’s choices.

### Conversations with their surgeon

Women indicated that they trusted the advice of the surgeon even more than other information sources. Conversations that women had with their surgeons were helpful in increasing their understanding of the surgical options and their confidence in the procedure. When women felt comfortable with their surgeon and their expertise, they were able to ask questions to clarify their options and felt confident in moving forward with the surgical option. Two women who opted for UPSs explained that because they ultimately trusted their surgeon when she said that the two approaches were equivalent in cure, they felt comfortable proceeding with a POP surgery that did not include a hysterectomy, noting that they “just trusted [the surgeon].”

Most women indicated that despite being told that both surgical options were equivalent in cure, that they still asked their surgeon for a recommendation between the two approaches. Women often asked female surgeons specifically what they would recommend “if they were me,” using that advice to make their decision. This behavior is another example of heuristics in gynecologic decision-making, and patients using a shortcut by asking the surgeon’s what they would do for themselves as a method of dealing with information that perhaps felt overly complex or voluminous.

## Discussion

This qualitative study describes the experiences of 26 women seeking surgical treatment for POP, finding that women’s decision-making for prolapse surgery is complex and not always based (solely or otherwise) on factual evidence presented to them during surgical consultation. Decisions were affected by individual’s perceptions of the risks and benefits of each approach (including potential recurrence of POP), their beliefs about which surgery would be best given their personal circumstances, traditional norms, and the recommendation of and information provided by their surgeon. Our results indicate that there is no single pathway for decision-making and no single set of factors determining women’s choice to remove or preserve their uterus.

This study adds to the small body of literature which has examined preferences regarding hysterectomy-based versus uterine-preserving surgery for treatment of POP, adding nuance to the existing literature. In a cross-sectional analysis of information priorities for decision-making regarding POP treatment (n = 100),^27^ the authors found that women viewed treatment success, potential complications and side effects, and quality of life after treatment as the most important factors to consider. Good et al.^28^ evaluated attitudes toward the uterus for women with POP, finding that women’s views on their uterus varied and that most women disagreed with statements indicating that the uterus was important for femininity and sense of self. Frick et al.^12^ echoed these findings, with women in their study reporting relatively neutral attitudes toward the uterus as being beneficial for sexuality or femininity, although 60% indicated they would choose to keep their uterus if an equally effective option was offered. Similar to qualitative work done regarding female incontinence,^29^ this study extends exploration of decision-making beyond clinical aspects to consider women’s thoughts about their uterus, societal ideals about which surgery is best, and women’s trust in their surgeon when deciding about POP surgical approach.

In contrast to other work where women do not receive information or choice around hysterectomy-based and uterine-preserving treatment,^8,22^ all women in this study were counseled about both techniques. Women indicated appreciation at having a choice to keep their uterus. However, despite attempts to standardize information and to clarify the clinical equipoise on the surgical approaches, women were sometimes left with a sense that one surgery is the “best” from their clinical encounters. This speaks to the persistent narrative that hysterectomy-based surgery is the best or default option for treating prolapsed,^30^ despite evidence that UPSs have equal, if not superior, outcomes.^10^

Women relied heavily on the recommendation of surgeons when deciding which surgery to have. It has been suggested that a surgeon’s own ability to offer UPS predicts whether uterine preservation is offered; however, in this study, all surgeons were trained in both approaches. Yet, participants reported numerous anecdotes that some surgeons implied a preference for hysterectomy when asked, “What would you do for yourself?.” Further work is required to understand why surgeons would subtly convey superiority of hysterectomy-based POP surgery. Possibilities include bias toward a traditional surgical viewpoint; resistance to adoption of recent scientific knowledge^31^; or financial considerations, given that in the setting where this study was conducted, removal of a uterus pays more than suspension. Ultimately, clinicians have a responsibility to ensure that women are only given information rooted in scientific evidence, as the well-documented power dynamic between clinician and patient^32^ is evident with women in this study reporting they trusted their surgeon as the best source of information, and some women posing personal questions to their female surgeon as a surgical decision-making short cut (heuristic).^33^ Surgeons should examine their own personal beliefs, especially as they pertain to the equipoise related to uterine preservation, to ensure they are presenting options free of unfounded bias.

The non-emergent nature of prolapse treatment leaves space for shared decision-making between woman and physician.^34^ True-shared decision-making necessitates women receiving information regarding all their treatment options, the respective risks and benefits, and the potential outcomes associated with each, as well as the support needed to engage with this information and evaluate it within their own values and individual circumstances. However, if women are not routinely offered UPS during clinical counseling, shared and informed decision-making cannot take place. Thus, surgeons lacking the skillset to perform UPS should still ensure that surgical options presented include procedures they themselves cannot perform and provide referral pathways to surgeons who can perform UPS if the patient prefers it. This is important not only for the shared decision-making process but also to recognize women’s bodily autonomy and their right to make informed decisions about their health.^35^ Without this, clinicians are continuing the worrisome trend in medicine of devaluing women’s bodily autonomy,^15^ ignoring their ability and right to make decisions regarding their own bodies,^21^ and adopting a paternalistic approach to decision-making which assumes that the doctor knows what is best for each woman.^36^

It is clear from our findings that, despite when standardized informational materials outlining the clinical equipoise between uterine-preserving and hysterectomy-based surgery and clinical counseling is provided to the women, misconceptions regarding the surgery options still exist (such as the personal belief that removal of uterus provides clinical benefit or that prolapse severity dictates the options for surgery). These misconceptions can have a significant impact on women’s decision-making and seemed to be more likely to push women toward hysterectomy-based surgeries, perhaps because of internalized ideas about prolapse treatment and different interpretations of the risks and benefits of each surgery. There is a need for clinicians to anticipate such misconceptions and be prepared to present information in a clear and value-free manner. This study has demonstrated the weight of surgeons’ voices for women, and as such, they should be cautious about what is conveyed from an anecdotal viewpoint. Given that the use of standardized patient pamphlets did not negate some women inferring superiority to a more invasive surgery, alternate forms of information delivery such as infographics and decision-aids should be evaluated for their ability to convey the complexities when considering the two approaches, potential trade-offs, and for their support of women’s decision-making processes in the context of their personal values, beliefs, and circumstances.

This study also demonstrated several interesting findings. First, women appeared to accept hysterectomy as the normative, default procedure. Second, both women selecting hysterectomy-based and uterine-preserving surgery expressed dismissive sentiments toward the uterus as “useless” after childbirth and that their surgeon should “get rid of it.” Taken together with the fact that many women had an understanding that hysterectomy was used to treat prolapse prior to their consultation, despite having only limited understanding of POP as a condition, these findings demonstrate how the historical patriarchal views of surgical gynecology have permeated broader society, leading women to accept hysterectomy as a default procedure in the field of gynecology.

This study had several limitations. Our sample consisted of primarily White, well-educated, heterosexual, cisgender women. It is possible that treatment decision-making for women of other races and ethnicities, sexual orientations, and gender identities, who may have unique experiences with marginalization in medicine and society more broadly, may be influenced by other factors. This study took place in a clinical center where women were presented with both surgical options during their initial surgical consultation; however, this may not always be the case. It is also possible that although women’s surgery choice did not differ by surgeon, given that proportions of uterine-preserving and hysterectomy-based surgery were similar for each, subtle differences in the way information was presented and received could have impacted individual choices. As well, although the interviewers were not involved in the surgical consultation, women may have felt like they were unable to critique the surgeons or clinical staff, which may have impacted their responses regarding their decision-making. Furthermore, it is possible that system-level influences specific to this study setting may have influenced women’s treatment decision-making around prolapse, and these types of barriers may differ across location and population. For example, at the time when this study was conducted, the patient-facing health information website provided by the Government of Alberta^37^ presented only information for the traditional, hysterectomy-based surgery as an option for POP. Given that women may seek information from a trusted party such as a government health authority, this could influence a societal viewpoint that hysterectomy-based surgery is superior, as well as create a barrier to women who felt strongly that they do not want to undergo hysterectomy, resulting in them not seeking treatment because uterine-preserving options are not presented as available.

Given the dearth of qualitative literature examining the experiences of women with POP and their decision-making between hysterectomy-based and uterine-preserving surgery for treatment of POP, our research adds women’s voices to the conversation and highlights their needs for information and conversation regarding treatment for POP. This study also heightens awareness of prolapse as a women’s health issue, helping to reduce the stigma and shame that women feel, normalizing treatment-seeking for POP, and conveying the need for information about POP to become normalized in discussions about women’s health.

## Conclusion

We examined women’s decision-making regarding hysterectomy-based versus uterine-preserving surgeries as treatment for POP. Women used both clinical and personal factors to make their decision, relying on the available evidence, their own personal values, and the recommendation of their surgeon, sometimes using the “default” or pushing for an opinion from their surgeon as a short cut to decision-making. Women demonstrated knowledge of hysterectomy’s use in POP surgery prior to consultation, but not uterine preservation. It is important to question high rates of hysterectomy in POP surgery as part of the intentional dismantling of patriarchal norms in women’s health, especially in the context of clinical equipoise. Given the need for women to be truly informed of their treatment options, clinicians should be prepared to offer both surgical options as treatment for POP, even if UPSs fall outside their skillset. Clinicians should also ensure that women receive adequate information and counseling regarding their surgery and should be prepared to pre-empt misconceptions that women may have regarding the procedures, as well as understand the role of women’s inherent values and perceived norms in the decision-making process.

## Supplemental Material

sj-docx-1-whe-10.1177_17455057231181015 – Supplemental material for Factors affecting women’s decision between uterine-preserving versus hysterectomy-based surgery for pelvic organ prolapse Click here for additional data file. Supplemental material, sj-docx-1-whe-10.1177_17455057231181015 for Factors affecting women’s decision between uterine-preserving versus hysterectomy-based surgery for pelvic organ prolapse by Kaylee Ramage, Ariel Ducey, Natalie V Scime, Erin Knox and Erin A Brennand in Women’s Health

sj-docx-2-whe-10.1177_17455057231181015 – Supplemental material for Factors affecting women’s decision between uterine-preserving versus hysterectomy-based surgery for pelvic organ prolapse Click here for additional data file. Supplemental material, sj-docx-2-whe-10.1177_17455057231181015 for Factors affecting women’s decision between uterine-preserving versus hysterectomy-based surgery for pelvic organ prolapse by Kaylee Ramage, Ariel Ducey, Natalie V Scime, Erin Knox and Erin A Brennand in Women’s Health

## Declarations

**Ethics approval and consent to participate:** Ethics approval for this study was received from University of Calgary’s Conjoint Health Research Ethics Board (REB19-2134); all participants provided written, informed consent.

**Consent for publication:** Informed consent to publish the research findings was obtained from participants.

**Availability of data and materials:** Not applicable.
