# Supplementary material for: Factors affecting women’s decision between uterine-preserving versus hysterectomy-based surgery for pelvic organ prolapse
Source: Womens Health (Lond). 2023 Jun 30;19:17455057231181015. doi: 10.1177/17455057231181015 (PMC10333630; doi:10.1177/17455057231181015)
Supplement: sj-docx-1-whe-10.1177_17455057231181015 – Supplemental material for Factors affecting women’s decision between uterine-preserving versus hysterectomy-based surgery for pelvic organ prolapse [file sj-docx-1-whe-10.1177_17455057231181015.docx]

**Interview Guide for Patients**

Much of the interview will be handled as a conversation, which moves back and forth between topics and reveals naturally occurring concerns, meanings and actions. Generic prompts will be used to elicit examples and stories (such as: “can you give an example?” “How did that happen?”)

*Purpose: to explore the woman’s experience of pelvic organ prolapse (POP), the social contexts in which she experiences POP, and her expectations of treatment.*

1. I understand you are scheduled to have surgery for pelvic organ prolapse with a surgeon at the Foothills Hospital. Could you describe how you came to this point?

[Will prompt for following specific information if not spontaneously included in narrative]

- What led you to seek treatment? In what ways has prolapse impacted you?
- Was your prolapse something you “discovered” on your own, or did a health professional diagnose it first and then tell you that you had it?
- What types of care providers have you consulted in the process? What did those care providers tell you about prolapse in general and how it can be treated?
- What was the length of time between first “discovering” your prolapse and seeing your surgeon for the first time? How long did you wait for surgery? Did you think that was a reasonable wait?
- What has the experience of the condition been like for you? How has it affected your daily life, including activities and relationships?
  - Has your condition had an impact on your intimate relationships or sex life?

1. Thinking back to before you had your first visit with your surgeon, do you remember having any thoughts or opinions about what would or should be done about your POP?

[Will prompt for following specific information if not spontaneously included in narrative]

- If the woman had some pre-existing thoughts/opinions, ask what experiences she thinks lead to those opinions. Examples can include a friend/family member having POP surgery, something another care provider said, something she read. Try pin down the source.
- Prior to that visit, were you specifically seeking surgery, or hoping to avoid it?

1. Prior to those medical visits, were you aware that sometimes the uterus is removed as part of surgery to repair prolapse?
2. Is this the decision you thought you were going to make before you saw the surgeon?
3. Please think back to the consultation and any follow-up visits you had with your surgical team. How did your consult with Surgeon X affect your thinking about your condition and its treatment? What did you take away from that consultation as the most important considerations?

[Will prompt for following specific information if not spontaneously included in narrative]

- Were the options of having your uterus removed (hysterectomy) versus having the uterus suspended in a thorough and clear manner? Were you left with any questions that were unanswered?
- Do you feel like any of the information the surgeon presented was conflicting? Or confusing?
- Were you surprised at all by the information they gave you?
- Do you feel like the way you were counselled was prescriptive – such as “you should do this….” – or were you the one to make the choice about hysterectomy vs. uterine suspension – such as “I will do either, whichever you would like”
- Do you feel like the surgical advice about what to do with your uterus was specifically tailored to you – such as “based on your PAP smear results, or menstrual pain, I would recommend XYZ”

1. What is your understanding of the treatment options – hysterectomy or uterine suspension as part of your prolapse repair - that are available to you? What do you understand as the pros and cons of these treatment options? Was one of the options – hysterectomy vs. uterine suspension – presented has having more or less surgical risk? More or less surgical success?
2. Is the surgical choice you made – hysterectomy or uterine suspension - the decision you thought you were going to make before you saw the surgeon?
3. Did the diagnosis of pelvic organ prolapse change how to you viewed yourself as a woman?
4. Would removal of your uterus as part of a surgical procedure change how you viewed yourself as a woman?

[Will prompt for following specific information if not spontaneously included in narrative]

- Do you feel as through removal of your uterus is inherently tied to your gender identity?
- If under age **45**, would loss of your reproductive potential through removal of the uterus change how you view your overall health or “womanhood”?
- If under age **50**, Does loss of your menstrual cycle through removal of your uterus change how you view your overall health or “womanhood”?

1. Have you talked to anyone other than your surgeon about this decision and did that play a part in your decision?

*Examples: family members, friends, co-workers, patient groups*

1. What other sorts of information have you consulted to try to understand your condition and treatment options? (Online sources, printed information/pamphlets, friends)
2. What do you hope the results of the surgery will be? What kind of results from the surgery will make it worth undergoing surgery, in your mind?
3. Based on your experience with POP, where should our research team plan to share the results of our work to better inform women about their surgical options before they see a surgeon?

Examples: patient advocacy groups, professional associations, lay media (get names of publications/magazines), blogs, social media – for example did they check to see if their own surgeon had a Twitter/IG/FB page for patients?
